# Lack of Phylogeographic Structure in the Freshwater Cyanobacterium *Microcystis aeruginosa* Suggests Global Dispersal

**DOI:** 10.1371/journal.pone.0019561

**Published:** 2011-05-05

**Authors:** Ineke van Gremberghe, Frederik Leliaert, Joachim Mergeay, Pieter Vanormelingen, Katleen Van der Gucht, Ann-Eline Debeer, Gissell Lacerot, Luc De Meester, Wim Vyverman

**Affiliations:** 1 Laboratory of Protistology and Aquatic Ecology, Ghent University, Ghent, Belgium; 2 Phycology Research Group, Ghent University, Ghent, Belgium; 3 Laboratory of Aquatic Ecology and Evolutionary Biology, Katholieke Universiteit Leuven, Leuven, Belgium; 4 Research Institute for Nature and Forest, Geraardsbergen, Belgium; 5 Department of Aquatic Ecology and Water Quality Management, Wageningen University, Wageningen, The Netherlands; 6 Facultad de Ciencias, Sección Limnología, Universidad de la República, Montevideo, Uruguay; Smithsonian Institution National Zoological Park, United States of America

## Abstract

**Background:**

Free-living microorganisms have long been assumed to have ubiquitous distributions with little biogeographic signature because they typically exhibit high dispersal potential and large population sizes. However, molecular data provide contrasting results and it is far from clear to what extent dispersal limitation determines geographic structuring of microbial populations. We aimed to determine biogeographical patterns of the bloom-forming freshwater cyanobacterium *Microcystis aeruginosa*. Being widely distributed on a global scale but patchily on a regional scale, this prokaryote is an ideal model organism to study microbial dispersal and biogeography.

**Methodology/Principal Findings:**

The phylogeography of *M. aeruginosa* was studied based on a dataset of 311 rDNA internal transcribed spacer (ITS) sequences sampled from six continents. Richness of ITS sequences was high (239 ITS types were detected). Genetic divergence among ITS types averaged 4% (maximum pairwise divergence was 13%). Preliminary analyses revealed nearly completely unresolved phylogenetic relationships and a lack of genetic structure among all sequences due to extensive homoplasy at multiple hypervariable sites. After correcting for this, still no clear phylogeographic structure was detected, and no pattern of isolation by distance was found on a global scale. Concomitantly, genetic differentiation among continents was marginal, whereas variation within continents was high and was mostly shared with all other continents. Similarly, no genetic structure across climate zones was detected.

**Conclusions/Significance:**

The high overall diversity and wide global distribution of common ITS types in combination with the lack of phylogeographic structure suggest that intercontinental dispersal of *M. aeruginosa* ITS types is not rare, and that this species might have a truly cosmopolitan distribution.

## Introduction

Dispersal, geographic isolation, past range restrictions and expansions, drift processes, founder events and selection all leave their signature in the lineage composition of contemporary populations [1]. The importance of such historical factors for free-living microbial organisms is contentious however, given that the large population sizes and high passive dispersal capacity of these organisms may drive a ubiquitous distribution with little or no biogeographic structuring [2], [3]. However, biogeographic and macro-ecological studies at the community level have shown that relatively few free-living microbial eukaryotes have cosmopolitan distributions [4], [5], [6], [7]. Many species show pronounced phylogeographic structure, or even regional or continental endemism, which counteracts the previously held paradigm of continuous and global panmixia.

Prokaryotes are generally smaller and have faster reproduction cycles than the eukaryotic microorganisms that were the subject of these biogeographic studies [8]. In addition, many bacteria have resistant dormant stages, or metabolic non-active cells that can survive passive transport through a hostile environmental landscape matrix [9]. Therefore, bacteria have been thought to experience virtually no dispersal limitation [3]. The absence of spatial structuring in bacterial communities has been corroborated by molecular data for soil [10], marine [11] and freshwater bacteria [12], [13] including cyanobacteria [14]. Conversely, several studies have reported clear phylogeographic structuring in other prokaryotes, including marine [15], soil [16] and soil-freshwater bacteria [17]. For prokaryotes occurring in extreme environments such as hot volcanic springs or deep-sea hydrothermal vents, phylogeographic structure indicates the effects of strong geographic isolation and dispersal constraints [18], [19], [20], although not all thermophylic (cyano)bacteria show clear spatial structure [21]. For more widely distributed bacteria, biogeographic patterns may result from historical (e.g. dispersal limitation) and/or contemporary environmental processes (e.g. local selection) [22], [23]. The relative importance of these processes in structuring microbial systems is still poorly understood [3]. Few studies have addressed questions of phylogeographic structure and dispersal limitation in bacteria on a truly global scale in discontinuous but globally common habitats, and yet such studies would provide a realistic insight into the degree of dispersal limitation typically encountered by bacteria.

The cyanobacterium *Microcystis* abounds in eutrophic and hypertrophic freshwater bodies worldwide [24], [25], [26], [27]. Such freshwater bodies are globally common and can be regarded as aquatic islands in a terrestrial and marine matrix. They are therefore ideally suited to study the role of dispersal limitation for free-living microorganisms [13]. As *Microcystis* often displays mass developments at the surface of lakes, it can easily be detected and sampled, and is therefore a good model organism to study global biogeographical patterns in free-living bacteria. *Microcystis* forms dense blooms that may be toxic, causing economical and ecological problems worldwide [28]. A better understanding of patterns of dispersal and genetic structure of *Microcystis* is therefore also relevant in the light of control of toxic cyanobacterial blooms.

*Microcystis* has a complex taxonomic history. The genus includes, next to the type species *M. aeruginosa*, a number of other species that have been delimited on the basis of colony morphology. These morphospecies, however, are not supported by molecular data forming a clade of nearly identical 16S rDNA sequences [29], [30]. Based on this extremely low 16S sequence divergence, along with DNA-DNA hybridisation data, Otsuka *et al.* [31], [32] suggested merging all morphospecies into a single species. We include various described morphospecies of *Microcystis* into our study, but these all refer to the name *M. aeruginosa* following Otsuka *et al.* [31].

On regional scales, significant spatial differences have been observed in *Microcystis* genotypic composition, yet phylogeographic structuring seemed absent [33], [34], [35]. On larger geographical scales, both presence [36], [37] or absence [34], [38], [39], [40], [41] of biogeographic structuring in *Microcystis* has been suggested depending on the sampled area or markers used. A global study of biogeographic patterns in relation to climatic conditions is still lacking.

The fast-evolving 16S–23S rDNA ITS region has proven to be a useful marker for phylogeographic studies of various (cyano)bacteria [40], [42], [43], [44], [45], [46]. In *Microcystis*, considerable ITS variation among *Microcystis* strains has been shown [34], [39]. Possibly, distinct ecotypes ( = physiologically different strains) may be distinguished in *Microcystis* by ITS sequencing as shown for the cyanobacterium *Prochlorococcus* [45], [47]. A link between ITS type and phenotypic and chemotypic traits was suggested for *Microcystis* [39], [42], [48], [49]. Additionally, van Gremberghe *et al.* [50] showed a (limited) environmental influence on *Microcystis* ITS population structure in Tigray (Ethiopia). The *Microcystis* genome contains two identical rRNA operons, although point mutations may occur occasionally [39], [42], [51]. Based on these criteria and the fact that a large number of ITS sequences is available in GenBank, ITS was selected as phylogenetic marker.

This study assesses global biogeographical patterns and dispersal of *M. aeruginosa* on six continents based on sequence variation.

## Methods

### Dataset construction

**Figure 1:**
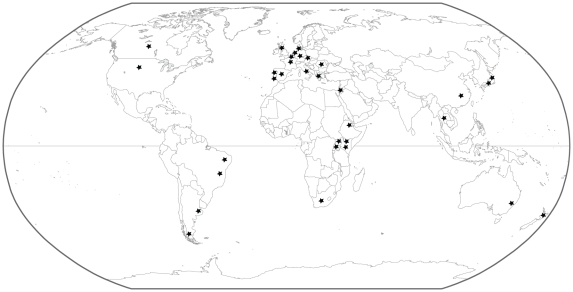
Map indicating the origin of the *Microcystis* ITS sequences used in this study.

Our dataset consists of 311 ITS sequences of *Microcystis* sampled from six continents: Europe (199), Africa (40), Asia (45), North America (7), South America (12) and Oceania (8). All sequences from Belgium (52), South America (12) and Ethiopia (29) were newly obtained in addition to some sequences from Denmark (5), The Netherlands (7) and Spain (2) (Table 1, Figure 1). New sequences were generated using three methods: by sequencing bands of Denaturing Gradient Gel Electrophoresis (DGGE) of water samples (57), by cloning of mixed PCR products from water samples (36), or by direct sequencing of isolated and cultured *Microcystis* strains (14). These were completed with 204 sequences from GenBank (http://www.ncbi.nlm.nih.gov/Genbank/), which were also obtained from cultivated isolates (135), sequenced DGGE bands (28) or cloned PCR fragments (41). An overview of all sequences used in this study is shown in Table S1. Newly generated sequences were deposited in GenBank under accession numbers HQ415607–HQ415713 (Table S1). Because no quantitative sequence data (i.e. abundance of particular ITS types per location) were available for most locations, only a single sequence of each ITS type per country was included in the dataset.

**Table 1:** Origin of the ITS sequences used to infer the global phylogeography of *Microcystis*.

Continent	Population	Climate	No. of sampled lakes	No. of distinct sequences
Europe	Belgium	Cfb	37	52
	The Netherlands	Cfb	10	38
	Germany*	Cfb	3	8
	Italy	Csa	1	3
	Spain	Csa	3	11
	Portugal-Csa	Csa	1	1
	Portugal-Csb	Csb	1	1
	Greece*	Csa	2	26
	Romania	Dfb	1	6
	France	Cfb	1	37
	Scotland*	Cfb	3	9
	Czech Republic	Cfb	1	1
	Denmark*	Cfb	5	6
Africa	Uganda-Af	Af	1	4
	Uganda-Aw*	Aw	2	3
	Kenya-Af*	Af	2	2
	Kenya-Aw	Aw	1	1
	Ethiopia*	BSh	29 (*: 5)	29
	South Africa	BSh	1	1
Asia	China*	Cfa	2	7
	Japan-Cfa*	Cfa	8 (*: 5)	26
	Japan-Dfb	Dfb	2	3
	Thailand*	Aw	3	7
	Israel	Csa	1	2
North America	Canada*	Dfc	1	2
	USA*	Dfb	4	5
South America	Brazil-Aw*	Aw	5	5
	Brazil-As*	As	2	3
	Argentina-ET*	ET	3	3
	Argentina-Cfc	Cfc	1	1
Oceania	New Zealand*	Cfb	5	6
	Australia	Cfb	2	2

Only the populations indicated with an asterisk were used in the restricted dataset correcting for differences in sample size (asterisk in the column ‘number of sampled lakes’ indicates the number of lakes selected in the restricted dataset). Climates were classified according to Köppen-Geiger. Main climates: A = equatorial, B = arid, C = warm temperate, D = snow, E = polar. Precipitation: S = steppe, f = fully humid, s = summer dry, w = winter dry. Temperature: a = hot summer, b = warm summer, c = cool summer, h = hot arid. T = polar tundra.

### Sampling, strain isolation and culture conditions

Water samples from lakes and ponds in Europe, South America and Ethiopia were filtered through a 25 mm 0.2 µm GSWP filter (Millipore) and immediately frozen at −20°C. Individual *Microcystis* colonies from samples from Belgium and Ethiopia were picked out using sterile glass Pasteur pipettes under a stereo microscope. The strains were grown in WC medium [52] (but without pH adjustment or addition of vitamins) at 19°C, an irradiance of approximately 30 µmol photons m^−2^ s^−1^ and a 12∶12 h light∶dark cycle. In total, ten ITS sequences from strains isolated from Belgium and two from Ethiopia were included in the molecular analyses.

### DNA extraction and PCR amplification

DNA from the water samples and isolated strains was extracted using bead beating, phenol extraction and ethanol precipitation as described by Zwart *et al.* [53]. After extraction, the DNA was purified on a Wizard column (Promega). Complete ITS sequences of the isolated *Microcystis* strains were amplified using the protocol described by Janse *et al.* [54] using the primers CSIF and ULR. For the DNA from the water samples, a specific nested-PCR protocol based on Janse *et al.* [54] was developed to amplify only *Microcystis* ITS sequences. In a first PCR, a specific 16S rDNA primer for *Microcystis* (CH) described by Rudi *et al.* [55] was used as forward primer combined with the universal reverse 23S rDNA primer ULR [54]. This PCR was performed using the protocol described by Janse *et al.* [54]. The resulting PCR product was purified using a QiaQuick PCR purification kit (QiaGen), diluted 10×, and used as template (2 µl in a total volume of 50 µl) for a second PCR with the cyanobacterium-specific 16S rDNA primer (GC)-CSIF (with GC-clamp for DGGE-analysis, without GC-clamp for cloning) in combination with the universal primer ULR. The composition of the reaction mix was the same as for the first PCR. The second PCR started with a denaturation step of 5 min at 94°C. After pre-incubation, 30 cycles were performed. Cycle step times were 1 min each for denaturation (94°C), annealing (65°C) and extension (72°C). A final extension step was performed for 30 min at 72°C.

### DGGE profiling

DGGE was essentially performed as described by Muyzer *et al.* [56]. The denaturing gradient contained 35–40% denaturant [100% denaturant corresponded to 7 M urea and 40% (v/v) formamide]. Electrophoresis was performed for 16 h at 75 V and the temperature was set at 60°C. Finally, the gels were stained with ethidium bromide and photographed on a UV transillumination table with a CCD camera. Next, a small piece of gel from the middle of the target band was excised from the DGGE gel and incubated in 50 µl sterile TE buffer (10 mM Tris, pH 7.6, 1 mM EDTA) for 24 h at 4°C. The eluent was then reamplified and purified on DGGE one or two times. The resulting PCR products were purified using a QiaQuick PCR purification kit (QiaGen). Sequencing was performed with the ABI-Prism sequencing kit and the resulting sequencing reaction products were analysed on an automatic sequencer (ABI-Prism 3100).

### Cloning

*Microcystis*-specific ITS sequences obtained from samples from Belgium and Ethiopia were ligated into pGEM®-T Easy Vectors (Promega), and transformed into competent *Escherichia coli* JM109 cells. The transformed cells were plated on Luria-Bertani (LB) plates containing 20 µg l^−1^ ampicillin, 20 µg l^−1^ X-Gal (5-bromo-4-chloro-3-indolyl-β-D-galactopyranoside) and 5 µg l^−1^ IPTG (isopropyl-β-D-thiogalactopyranoside) and incubated overnight at 37°C. White recombinants were picked out and grown overnight in ampicillin-supplemented liquid medium (Luria-Bertani-Broth). The clones were screened for inserts using primers CSIF and ULR (see before). 40–70 clones per sample were screened by DGGE analysis (see before) to identify groups of clones containing (presumably) the same inserts. One or more representatives of each group were then chosen for sequencing. In total, 34 ITS sequences from Belgium and four from Ethiopia were obtained in this way. Sequencing was performed with the ABI-Prism sequencing kit and the resulting sequencing reaction products were analysed on an automatic sequencer (ABI-Prism 3100).

### Phylogenetic analysis

The 311 ITS sequences were aligned using MUSCLE [57] and manually adjusted (Dataset S1). The amount of phylogenetic signal versus noise in the alignment was assessed by two different approaches. First, the I_ss_ statistic, a measure of substitution saturation in molecular phylogenetic data sets, was calculated with DAMBE [58]. Second, the measure of skewness [g1-value calculated by using 10,000 randomly selected trees in PAUP* 4.0b10 [59] was compared with the empirical threshold values in Hillis & Huelsenbeck [60] to verify for non-random structuring of the data. Visual inspection of the alignment suggested the presence of multiple hypervariable regions in the ITS region and detailed analysis of DNA site polymorphism using DnaSP 4.50.3 [61] by means of the sliding window option (window size = 1, step size = 1) revealed eight hypervariable regions (see Results for details). To test whether these regions were phylogenetically informative, linkage between these hypervariable regions was assessed using Genetix v. 4.5 [62] by encoding each variant of a particular hypervariable site or region as a distinct allele and testing the Black & Krafsur [63] correlation coefficient for linkage disequilibrium. For this test to run, haploid data were considered as homozygous diploid data as suggested by Goudet [64]. Although this test is usually used to test for independence of inheritance of supposedly physically unlinked genetic markers, we used it to test the degree of correlation between proximate regions within the rDNA ITS locus (<300 base pairs apart). The hypervariable regions showed weak linkage, despite their close proximity (see Results). Because the hypervariable regions would potentially mask phylogenetic signals due to extensive homoplasy [65], we opted for performing subsequent analyses on the complete ITS dataset (from here on referred to as full dataset), as well as on the ITS alignment excluding the hypervariable regions (from here on referred to as stripped dataset).

Statistical parsimony networks [66] were constructed with TCS 1.21 [67], with calculated maximum connection steps at 95% and alignment gaps treated as missing data. Additionally, statistical parsimony analyses were performed using the hypervariable regions only (separate networks for each hypervariable region of more than two basepairs or one network for all hypervariable regions concatenated) to check for the presence of a phylogenetic and phylogeographical signal in these regions.

The production of PCR artefacts (e.g. chimeras and heteroduplexes) is a potential risk when mixed templates of related sequences are amplified by PCR [68], [69], and this would lead to an overestimation of the genetic variation through the amplification of artificial sequences [70], [71]. DGGE and cloning using PCR-amplified DNA obtained from water samples may involve such risks and therefore phylogenetic analyses were also performed on sequences derived from isolated strains only (149 sequences in total).

### RNA secondary structure analysis

Prediction of the RNA secondary structure of the ITS region was based on the complete rrn operon sequence extracted from the complete genome of *Microcystis* (strain NIES843, EMBL accession number AP009552; ITS sequence identical to BG08 of the present study). RNA sequences were folded using Mfold [72]. Foldings were conducted at 25°C using a search within 10% of thermodynamic suboptimality and the obtained RNA structures were compared with published data [47], [73]. The secondary structure diagram was created using RnaViz [74].

### Biogeographic and climatic structure analysis

The sequence dataset was divided into 32 pre-defined populations, each population consisting of sequences from a single country (Table 1). For each ITS sequence, the climate of the region of origin was indicated according to Köppen-Geiger [75]. For some larger countries spanning different climate zones, more than one population was considered. In total, twelve distinct climates were distinguished (Table 1). Since only a single sequence of each ITS type was included for each country in the dataset, the genetic diversity within these populations is overestimated. Therefore, we did not interpret the genetic diversity within the populations (see Results).

To test whether genetic distance was correlated to geographical distance (Isolation By Distance) the program IBDWS [76] was used. Nonparametric Mantel tests were performed for the full and stripped dataset to test for non random associations between matrices of genetic distances between all population pairs and matrices of pairwise geographical distances. Genetic distances were computed using Slatkin's [77] similarity measure: M = ((1/F_st_)−1)/4. Geographical distances between the different populations were measured using geographical coordinates of the lake from which the sequences were obtained or if several lakes were sampled per population, the centroid between these lakes was taken. We calculated the great-circle distances as well as the shortest distance over land since *Microcystis* might disperse easier over land using stepping stones in between sampled locations (lakes, ponds, pools) than across oceans. The latter distances were calculated using Google Earth (http://earth.google.com/). Mantel tests were performed using both types of geographical distances.

Patterns of genetic structuring based on the stripped ITS sequence alignment among geographical localities (continents) and climates were estimated by analysis of molecular variance (AMOVA) using Arlequin 3.1 [78]. AMOVA was performed only on the stripped dataset as the genetic diversity (number of ITS types) was too high to obtain reliable estimates in the full dataset. For inter-continental comparisons, using the total dataset can give a biased view as more populations were sampled in Europe compared to the other continents. To obtain comparable sample sizes, populations were selected based on the number of lakes sampled per country (2–5 lakes per country, except for Canada, see Table 1). AMOVA was also performed on the dataset containing all populations. In addition, we divided the selected dataset into two “super continents”: Afro-Eurasia (random selection of four populations: Greece, Denmark, Ethiopia and Japan) and the Americas (North and South America). AMOVA was also performed on the dataset containing all populations. Furthermore, three climate groups were considered. First, a group based on the climate according to Köppen-Geiger resulted in twelve different climates. Second, only climates represented by more than ten ITS types (Aw, BSh, Cfb, Csa, Cfa, Dfb) were considered. Third, the dataset was split into cold-temperate (Cfb, Dfc, Cfc, Csb, Dfb, ET) and (sub)tropical climate (Af, As, Aw, Cfa, Csa, BSh), based on the average year temperature (see legend table 1 for explanation of the codes of the different climates). To perform AMOVA, a distance matrix was calculated using Tamura & Nei distances [79] and significance levels were determined with 1000 permutations.

### Demographic analyses

The historical demographic structure of the genetic variation at the ITS locus was investigated using Tajima's *D* [80] and Fu's *Fs* [81] in DnaSP 4.5.03 [61]. Negative values of both test statistics result from purifying selection in a population at mutation-drift equilibrium, or from deviations from mutation-drift equilibrium that are due to population expansion events. We used these statistics to test for demographic expansions. Since these tests rely on the assumption that all nucleotide positions are equally mutable, we only performed them on the stripped dataset.

## Results

### Data exploration

**Figure 2:**
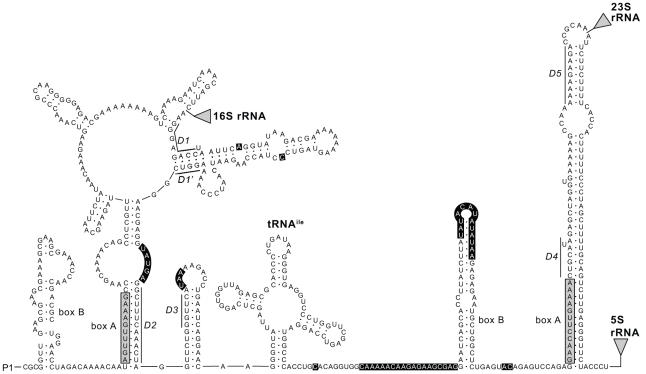
Predicted secondary structure of the spacer region of the rrn operon transcript. The rrn operon transcript shown in the figure is the 5′ leader sequence upstream from the 16S rRNA, the 16S–23S rRNA ITS and 23S-5S rRNA spacer of *Microcystis* strain NIES843 (EMBL accession number AP009552, ITS sequence identical to BG08 of the present study). P1 indicates the position of the promotor. Locations of the 16S rRNA, 23S rRNA and 5S rRNA are represented by triangles. The antiterminator box B stems and box A sequences are indicated in the leader and spacer domains, in accordance with Rocap *et al.* [47]. The conserved motifs (D1–D5, defined by Iteman *et al.* [73]) are marked. Positions of the hypervariable regions, identified in the present study, are indicated by a black background.

Specifications of the full and stripped ITS sequence alignments are given in Table 2. Maximum pairwise sequence divergence was 13% for the full and 5% for the stripped alignment. Although the I_ss_ statistic did not reveal significant saturation of the full alignment, the measure of skewness equalled the empirical threshold values in Hillis & Huelsenbeck [60], indicating that the alignment was only slightly more structured than random data. Analysis of DNA site polymorphism revealed eight hypervariable regions or positions in the ITS sequences that displayed extremely high nucleotide diversity (0.55>π>0.30, at positions 18, 44, 81–87; 114–116, 217, 226–248, 269–284, 306–307) compared to the average (0.03). RNA secondary structure analysis revealed that these regions were all positioned in loop regions (Figure 2). Sequencing artefacts could be excluded based on the observation that similar or identical mutations were observed for all regions in all sequences, irrespective of the method used for obtaining the sequences. Such variable regions would only be phylogenetically informative if their different genetic variants were autapomorphies, and if they were only transferred vertically and did not suffer from recombination. Under this model of inheritance, we expected particular variants of these mutation-prone regions to be highly correlated to each other (r>0.90) due to nearly complete physical genetic linkage. In the opposite case, these hypervariable sites or regions could behave as more independent units, meaning that associations with other hypervariable regions would be weaker. This lack of linkage and high level of homoplasy would increase the level of noise in the phylogenetic signal. All the aforementioned hypervariable regions showed significant but very weak linkage (r = 0.07–0.22; p<0.05), showing that these regions display incongruent phylogenetic signals in the large majority of cases. These regions did not conform to the assumption of equal mutation rates throughout the sequence according to a certain model of mutations. Most of these regions represented insertions/deletions of multiple nucleotides, thereby not conforming to classical single-step mutation models. They likely represent another process than the regular mutational processes in DNA. Because these regions potentially blur genetic signals in the rest of the data, further analyses on the genetic relationships between ITS sequences were performed on the complete ITS dataset, as well as on the dataset from which these hypervariable regions were excluded (stripped dataset). Removal of the hypervariable regions resulted in an alignment that was significantly more structured than random data, as indicated by the measure of skewness (Table 2).

**Table 2:** Specification of the full and stripped ITS sequence alignment and summary of models and model parameters obtained.

	ITS sequence alignment	
	Full	Stripped
Alignment length/variable sites/parsimony informative sites (basepairs)	374/142/68	314/99/33
Number of ITS types in the total dataset/total number of sequences	239/311	119/311
Number of ITS types per continent/total number of sequences per continent:		
Europe	162/199	86/199
Africa	34/40	26/40
Asia	41/45	21/45
North America	7/7	2/7
South America	10/12	7/12
Oceania	7/8	7/8
Number of ITS types detected once/number of ITS types detected more than once in the total dataset	205/34	94/25
Uncorrected pairwise sequence divergence (max/average %)	13/4	5/1
Measure of skewness (g_1_-value)	−0.079	−0.305
I_ss_ statistic (I_ss_/I_ss_.c, *p*-value of 32 taxon data subsets)	0.248/0.682, *p*<0.001	0.044/0.675, *p*<0.001

### Statistical parsimony analysis

**Figure 3:**
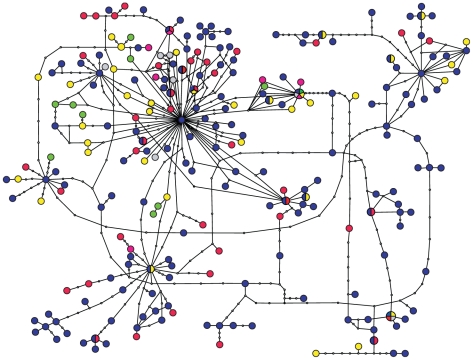
95% probability parsimony network of *Microcystis* rDNA ITS sequences based on the full ITS alignment. Colours indicate region of origin: blue: Europe, yellow: Africa, red: Asia, green: South America, grey: North America, pink: Oceania. A line between ITS types represents one mutational step, open circles represent ITS types not present in the sample.

Statistical parsimony analysis of the full ITS alignment resulted in a single, highly interconnected and largely unresolved network with maximum connection limit of 9 steps (Figure 3). Similar results were obtained when using sequences from isolated strains only, indicating that these results were not a consequence of PCR artefacts (data not shown). In total, the dataset contained 239 ITS types (gaps treated as missing), of which 34 were detected more than once (Table 2). Seventeen ITS types were detected on more than one continent, of which thirteen were found on two continents, two on three continents, one on four continents and one on five continents. A high diversity of ITS types was detected in each continent (Table 2). Parsimony networks based on the separate hypervariable regions were highly unresolved and showed a similar lack of geographic structuring (data not shown).

**Figure 4:**
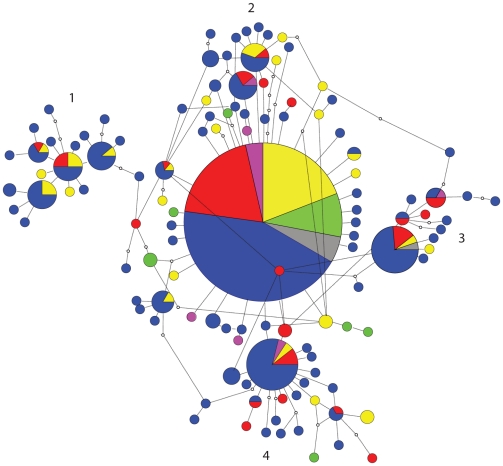
95% probability parsimony network of *Microcystis* rDNA ITS sequences based on the stripped ITS alignment. Colours indicate region of origin: blue: Europe, yellow: Africa, red: Asia, green: South America, grey: North America, pink: Oceania. A line between ITS types represents one mutational step, open circles represent ITS types not present in the sample. Radius of the circles represent number of sequences, numbers 1–4 indicate subclusters.

The parsimony network derived from the stripped ITS sequence alignment was to a certain extent still unresolved, but with a much lower degree of connection uncertainties (Figure 4). One particular ITS type accounted for fifteen percent of all sequences. This ITS type was also considered as the most likely root of the network by TCS, following the general trend that ancestral types tend to be centrally placed, are abundant and have many closely related derivates [82], [83]. This central type was distributed over all six sampled continents. Four smaller clusters (numbered 1–4 in Figure 4) could be discriminated, connected to this central group by one to five mutational steps, each of which contained sequences from two to four continents. The sequences from North and South America and Oceania were mostly grouped around the ancestral ITS type, but few sequences were available from these continents. The overall pattern suggests no relation between geographic origin and position in the network. In total, 119 stripped ITS types were detected (gaps treated as missing), of which 25 were detected more than once (Table 2). Sixteen ITS types occurred on more than one continent, of which seven occurred on two continents, six on three continents, two on four continents and one on six continents. The unique ITS types (detected only once) were generally connected to more frequently observed ITS types by a single mutational step (Figure 4). A high diversity of stripped ITS types was detected in each continent (Table 2). Twenty-two stripped ITS types were found in different climate regimes, of which eighteen were detected in (sub)tropical as well as cold-temperate climate regimes. Eleven different climates of a total of twelve were represented by the one dominant ITS type (only Csb was not included, however this climate was represented by only one sequence in the dataset). No genetic structuring based on temperature seemed present since the African sequences (warm climates) and European sequences (temperate climates) were spread all over the network.

### Isolation by Distance

A Mantel test for matrix correlation between genetic similarity (M) and geographic distance using great circle distances showed no significant negative correlation for the full dataset (*Z* = 28*10^11^, *r* = 0.127, one-sided *p* = 0.958) or the stripped dataset (*Z* = 16*10^11^, *r* = 0.131, one-sided *p* = 0.948). Similarly, a Mantel test for matrix correlation between genetic similarity (M) and geographic distance using the shortest distance over land showed no significant negative correlation for the full dataset (*Z* = 46*10^11^, *r* = 0.112, one-sided *p* = 0.918) or the stripped dataset (*Z* = 24*10^11^, *r* = 0.058, one-sided *p* = 0.775).

### Analysis of Molecular Variance (AMOVA)

AMOVA based on the stripped ITS sequence alignment showed no genetic structuring among continents (Table 3). The dominant component of genetic variation was found within continents (96.08%) instead of between them (3.92%). AMOVA on the dataset containing all populations showed similar results (not shown). A significant but weak genetic structure was found between the super continents Afro-Eurasia and America (*F_CT_* = 0.064, *p* = 0.01), but the dominant component of genetic variation was again found within (93.55%) instead of between these super continents (6.45%). AMOVA on the dataset containing all populations showed similar results (not shown). No significant genetic structuring was found among all twelve climate groups, among the six climates represented by more than ten ITS types, or between cold-temperate and (sub)tropical climates (Table 3).

**Table 3:** Results of AMOVA based on the stripped ITS sequence alignment.

	*F_CT_*	*p*
Continent	0.039	0.054
Afro-Eurasia/the Americas	0.064	0.010
Climate (all 12)	−0.003	0.654
Climate (6 most sampled)	0.001	0.423
Cold-temperate/(sub)tropical	−0.005	0.823

### Demographic analyses

Both Tajima's test (*D* = −2.393, *p*<0.01) and Fu's test (*Fs* = −127.581, *p*<0.0001) are indicative of a recent global expansion of *M. aeruginosa*. Note that the term ‘recent’ only has a qualitative meaning, relative to the rate of mutation and genetic drift at the ITS locus.

## Discussion

In this study, we analyzed biogeographical patterns and inferred dispersal patterns of the freshwater cyanobacterium *Microcystis aeruginosa* on a global scale using rDNA ITS sequence data sampled from six continents. The combination of a high genetic resolution with global sampling allowed a sound evaluation of the global biogeography of this cyanobacterial species. Admittedly, only one locus was used, but the lack of structure that we found can hardly be attributed to insufficient genetic variability.

Our results show that there are no genetically distinct clades of ITS sequences within the genus *Microcystis* and that genetic divergence is low compared to other cyanobacterial genera [47], [84]. This reinforces the hypothesis that all described taxa in the genus *Microcystis* represent a single homogenous bacterial taxon, *M. aeruginosa*, with a relatively young evolutionary history [31], [32]. This contrasts with ITS-based phylogenies of several other bacterial taxa in which, typically, well-supported and relatively deep evolutionary lineages are distinguished. For example, in the marine cyanobacterial genera *Synechococcus* and *Prochlorococcus*, genetically distinct ITS clades were detected, with very broad distributions [47], [84]. In these cyanobacteria, ITS diversity was comparable with *Microcystis*, however, genetic structure was clearly more pronounced.

Several regions in the ITS alignment were highly variable with only weak correlation between the various alleles of each locus, even among those that were separated by just a few dozen nucleotides. This lack of linkage and high level of homoplasy is very remarkable. A possible but scarcely conclusive explanation for this singularity is that it is the result of recombination within the genome itself. Several studies have revealed exchange of genetic information through horizontal gene transfer and recombination in cyanobacteria [85], [86], [87], [88]. Recently, Tanabe *et al.* [89] suggested that recombination is an important evolutionary force for the generation and maintenance of genetic diversity in *Microcystis*. Although there are many tests for recombination, they also rely on substitution rate homogeneity, and cannot distinguish autocorrelation in substitution rates among sites (such as found in mutation motifs, or these caused by secondary RNA or DNA structure) from true recombination [90]. As the observed hypervariable regions were all located in regions with higher expected mutation rates (loops), and therefore likely violate the assumption of substitution rate homogeneity, tests for recombination cannot provide reliable answers for the problems caused by the hypervariable regions. Kaneko *et al.* [91] and Frangeul *et al.* [51] showed that the genome of *Microcystis aeruginosa* is very plastic and displays a high transposon activity. The hypervariable regions found in the rDNA ITS region are too short to be transposons, however, and the exact nature of these regions remains uncertain. Notwithstanding these uncertainties, all analyses on the geographic genetic structure yielded concordant results.

The global distribution of *Microcystis*, along with the lack of geographical genetic structuring and the prevalence of a single, widespread ITS type (excluding the hypervariable regions) may be explained by two radically different scenarios: 1) *Microcystis* represents an ancient lineage, which acquired its worldwide distribution gradually on evolutionary time scales, or 2) *Microcystis* represents a young clade that spread globally recently, experiencing ongoing passive dispersal. Several clues favour the second hypothesis. Firstly, 16S rDNA data clearly indicates that *Microcystis* forms a distinct clade of nearly identical sequences, indicating a relative recent origin [29], [30]. Also, variation in ITS sequences is relatively low, supporting the 16S-based hypothesis of a young clade. Secondly, an ancient and slowly dispersing taxon would likely lead to isolated populations over long periods of time, which would leave a biogeographical signature (i.e. the presence of distinct, geographically isolated lineages or clear biogeographic structure). In contrast, our statistical parsimony analysis revealed a single, highly interconnected network, indicating little phylogeographic signal, which can be explained by current dispersal events. Thirdly, the test results of deviations from mutation-drift equilibrium (Tajima's *D*, Fu's *Fs*) support the hypothesis of a recent worldwide population expansion following a bottleneck or a selective sweep. Fourthly, the isolation-by-distance analysis showed no correlation between genetic and geographical distance, and analysis of molecular variance shows a lack of overall genetic differentiation between continents reinforcing the notion of frequent or occasional ongoing global dispersal of *Microcystis*. We only found a subtle geographic structure between Afro-Eurasia and the Americas, and ITS types from the Americas were absent in some of the subclusters in the stripped ITS network. However, this could be due to relatively low sampling in the Americas. It is difficult to compare the *F_CT_* values from the AMOVA directly with studies on other organisms as other molecular markers are often used. However, the finding that the dominant component of genetic variation was found within, instead of between continents, supports the ubiquitous nature of *M. aeruginosa*.

The exact temporal scale of dispersal remains uncertain due to the absence of an evolutionary timeframe of cyanobacteria, as well as limits of the design of the study and the temporal resolution that is inherent to the mutation rate of the studied ITS fragment. We can only state that the elapsed time since the most recent global dispersal events is probably not longer than the average time needed for new mutants to arise in isolated populations and increase above the detection threshold. This, in turn, depends on the population biology of *Microcystis*, the role of random drift versus selection and the mutation rate of the ITS sequence. Answers to these questions would require genetic markers with higher resolution as well as a spatially explicit experimental design to study colonization and succession dynamics. So far, the question remains whether the lack of geographic structure in the data is due to frequent global dispersal and gene flow among distant populations, or whether this is due to occasional transoceanic colonisations of new habitat patches combined with persistent founder effects. Population-genetic theory strongly argues against the first scenario, as a result of extremely large population sizes of bacterial populations that would necessitate large amounts of gene flow for immigrant strains to be detected in established populations and contribute to genetic differentiation [92]. It is therefore more plausible that the genetic structure of *Microcystis* populations is driven by founder effects that arise whenever new habitat patches are created [93], which are then colonized by a random selection of strains from regional or possibly global sources.

The absence of phylogeographic patterns found in this study is remarkable given the fact that freshwater bodies are small and often highly isolated habitat patches in a vast terrestrial matrix, which in itself is separated by extended oceanic regions at a global scale. The pattern in *M. aeruginosa* contrasts with that of another bloom-forming freshwater cyanobacterium, *Cylindrospermopsis raciborskii*, in which phylogenetic analyses based on four genes, including ITS, showed a clear clustering of strains according to continent [44]. Similarly, phylogeographic structure has been implied for the freshwater cyanobacterium *Arthrospira sp.*, based on the appearance of two discrete ITS clusters corresponding to Africa-Asia and America [46]. Contrary to *Microcystis*, *Cylindrospermopsis* and *Arthrospira* have a narrower ecological amplitude, mainly occurring in tropical to warm-temperate regions [94], [95], [96], [97]. The lack of intermediate suitable stepping stones in temperate regions may explain their more restricted dispersal, but local adaptation may be equally important. This illustrates that freshwater cyanobacteria can have different biogeographies, ranging from cosmopolitan to geographically restricted. Previous studies have also indicated a diversity in bacterial biogeographical patterns at different taxonomic levels [3], [22], [23], [98]. Although analyses of a single fast-evolving marker such as ITS provide a relatively fine level of genetic resolution, multi-locus analyses would offer additional insights into the behavior of microbial populations (cfr. the frequency of genetic exchange, recombination and lateral gene transfer).

Little information is available on the dispersal ability and transport mechanisms of *Microcystis* [99], [100]. *Microcystis* cells have thick cell walls and are grouped into colonies surrounded by a protective mucilage layer, which may protect them from harsh environmental conditions during dispersal. They have been observed in vegetative condition in the sediment [36], [101], and are tolerant to low temperatures and darkness [102]. The latter observations indicate that they might resist drought or intestinal conditions for a certain period, which could explain their ability for rapid global dispersal [100], [103]. The probability of successful passive long-distance dispersal depends strongly on the effectiveness of the carrier (animal or airborne dispersal), and the ability of *Microcystis* to tolerate the transport conditions [104], [105]. An effective way for *Microcystis* to disperse might be by migrating birds [97], [106]. During *Microcystis* blooms, colonies may easily be transferred by birds either through internal or external transport. If migrating birds were the dominant dispersal vectors, however, much stronger phylogeographic patterns could be expected, reflecting the north-south migration routes of water birds. The absence of such pattern suggests that transport may also be driven by other factors, such as wind dispersal. Transcontinental wind and dust events have been proposed to form atmospheric bridges over land and sea, facilitating long-distance dispersal of micro-organisms [107]. Finally, the worldwide occurrence of *Microcystis* strains might also be facilitated by human-mediated dispersal, mainly by the recreational use of water bodies and transport of fish and macrophytes, as already suggested for several other small aquatic organisms [108], [109].

No genetic structuring according to climate conditions was found in *Microcystis*, which contrasts with some other studies on cyanobacteria [14]. Several *Microcystis* ITS types were detected in a wide range of climates, indicating a broad tolerance or capacity for rapid local adaptation to different climatic conditions. We did not find indications that distinct ecotypes of *Microcystis* can be differentiated by ITS as has been suggested for *Prochlorococcus* [45], [47], but more research should confirm this. *Microcystis* populations are likely phenotypically and/or ecologically differentiated by different suites of functional genes. Indeed, a high functional diversity (cfr. differences in growth rate, colony formation, cell size and microcystin production) in sympatric and allopatric *Microcystis* strains has been shown by laboratory experiments [110], [111]. A number of studies have highlighted the extent of bacterial functional diversity and environmental adaptation, beyond the resolution of ribosomal DNA markers [112], [113], [114], [115]. The plasticity of the genome of *M. aeruginosa* [51], [89], [91] may be a key feature in allowing rapid local adaptation in a wide array of environmental conditions by yielding new interactions between genetic loci and therefore releasing additive genetic variance on which natural selection can act. In addition, also *de novo* mutations might be involved in rapid adaptation to novel environments given the large population sizes and short generation time of *M. aeruginosa*.

## Supporting Information

Dataset S1 rDNA ITS dataset with indication of ITS, tRNA and hypervariable regions (x).
(TXT)

Table S1 Overview of the sequences used in this study.
(DOC)
