# Supplementary material for: Lack of Phylogeographic Structure in the Freshwater Cyanobacterium Microcystis aeruginosa Suggests Global Dispersal
Source: PLoS One. 2011 May 5;6(5):e19561. doi: 10.1371/journal.pone.0019561 (PMC3088681; doi:10.1371/journal.pone.0019561)
Supplement: Table S1 — Overview of the sequences used in this study. (DOC) [file pone.0019561.s002.doc]

Table S1: Overview of the sequences used in this study

| **Accession number*** | **Country** | **Climate** | **Method** |
| --- | --- | --- | --- |
| AB015357 | China | Cfa | isolate |
| AB015359 | Thailand | Aw | isolate |
| AB015360 | Thailand | Aw | isolate |
| AB015363 | Japan | Cfa | isolate |
| AB015365 | Japan | Cfa | isolate |
| AB015366 | Japan | Cfa | isolate |
| AB015367 | Japan | Dfb | isolate |
| AB015368 | Japan | Dfb | isolate |
| AB015369 | Japan | Cfa | isolate |
| AB015370 | Japan | Cfa | isolate |
| AB015371 | Japan | Cfa | isolate |
| AB015372 | Thailand | Aw | isolate |
| AB015374 | Japan | Cfa | isolate |
| AB015375 | Japan | Cfa | isolate |
| AB015377 | Scotland | Cfb | isolate |
| AB015378 | China | Cfa | isolate |
| AB015379 | Thailand | Aw | isolate |
| AB015381 | China | Cfa | isolate |
| AB015383 | China | Cfa | isolate |
| AB015384 | Thailand | Aw | isolate |
| AB015385 | Thailand | Aw | isolate |
| AB015386 | Thailand | Aw | isolate |
| AB015387 | Japan | Cfa | isolate |
| AB015392 | China | Cfa | isolate |
| AB015393 | Japan | Cfa | isolate |
| AB254408 | Japan | Cfa | isolate |
| AB254409 | Japan | Cfa | isolate |
| AB254410 | Japan | Cfa | isolate |
| AB254411 | Japan | Cfa | isolate |
| AB254412 | Japan | Cfa | isolate |
| AB254413 | Japan | Cfa | isolate |
| AB254414 | Japan | Cfa | isolate |
| AB254415 | Japan | Cfa | isolate |
| AB254416 | Israel | Csa | isolate |
| AB254417 | Israel | Csa | isolate |
| AB254438 | Japan | Cfa | isolate |
| AB254439 | Japan | Cfa | isolate |
| AB254440 | Japan | Cfa | isolate |
| AB254444 | Japan | Cfa | isolate |
| AB254445 | Japan | Cfa | isolate |
| AB333807 | Japan | Dfb | isolate |
| AB333809 | Japan | Cfa | isolate |
| AB333815 | Japan | Cfa | isolate |
| AB333817 | Japan | Cfa | isolate |
| AF387609 | Canada | Dfc | isolate |
| AJ605140 | The Netherlands | Cfb | isolate |
| AJ605141 | The Netherlands | Cfb | isolate |
| AJ605142 | The Netherlands | Cfb | isolate |
| AJ605143 | The Netherlands | Cfb | isolate |
| AJ605145 | Scotland | Cfb | isolate |
| AJ605146 | The Netherlands | Cfb | isolate |
| AJ605150 | The Netherlands | Cfb | isolate |
| AJ605155 | Portugal | Csb | isolate |
| AJ605156 | The Netherlands | Cfb | isolate |
| AJ605159 | Italy | Csa | isolate |
| AJ605164 | Czech Republic | Cfb | isolate |
| AJ605165 | Germany | Cfb | isolate |
| AJ605166 | The Netherlands | Cfb | isolate |
| AJ605169 | Germany | Cfb | isolate |
| AJ605170 | The Netherlands | Cfb | isolate |
| AJ605172 | Germany | Cfb | isolate |
| AJ605173 | The Netherlands | Cfb | isolate |
| AJ605174 | Scotland | Cfb | isolate |
| AJ605175 | Denmark | Cfb | isolate |
| AJ605177 | The Netherlands | Cfb | isolate |
| AJ605180 | The Netherlands | Cfb | isolate |
| AJ605181 | The Netherlands | Cfb | isolate |
| AJ605182 | Germany | Cfb | isolate |
| AJ605183 | Germany | Cfb | isolate |
| AJ605184 | The Netherlands | Cfb | isolate |
| AJ605185 | The Netherlands | Cfb | isolate |
| AJ605186 | The Netherlands | Cfb | isolate |
| AJ605187 | Portugal | Csa | isolate |
| AJ605188 | The Netherlands | Cfb | isolate |
| AJ605189 | Scotland | Cfb | isolate |
| AJ605190 | The Netherlands | Cfb | isolate |
| AJ605191 | Scotland | Cfb | isolate |
| AJ605193 | The Netherlands | Cfb | isolate |
| AJ605194 | Italy | Csa | isolate |
| AJ605195 | The Netherlands | Cfb | isolate |
| AJ605196 | The Netherlands | Cfb | isolate |
| AJ605197 | Scotland | Cfb | isolate |
| AJ605199 | The Netherlands | Cfb | isolate |
| AJ605200 | Germany | Cfb | isolate |
| AJ605201 | The Netherlands | Cfb | isolate |
| AJ605202 | The Netherlands | Cfb | isolate |
| AJ605203 | Scotland | Cfb | isolate |
| AJ605204 | Germany | Cfb | isolate |
| AJ605205 | Scotland | Cfb | isolate |
| AJ605206 | Scotland | Cfb | isolate |
| AJ605207 | The Netherlands | Cfb | isolate |
| AJ605208 | The Netherlands | Cfb | isolate |
| AJ605210 | Italy | Csa | isolate |
| AJ605211 | Germany | Cfb | isolate |
| AM235780 | The Netherlands | Cfb | isolate |
| AM235781 | The Netherlands | Cfb | isolate |
| AM235782 | The Netherlands | Cfb | isolate |
| AM421549 | Uganda | Af | isolate |
| AM421552 | Uganda | Af | isolate |
| AM421556 | Uganda | Af | isolate |
| AM421557 | Uganda | Af | isolate |
| AM421559 | Uganda | Aw | isolate |
| AM421560 | Uganda | Aw | isolate |
| AM421562 | Uganda | Aw | isolate |
| AM421568 | Kenya | Af | isolate |
| AM421569 | Kenya | Af | isolate |
| AM421572 | Kenya | Aw | isolate |
| AY266124 | Spain | Csa | isolate |
| AY266126 | Spain | Csa | isolate |
| AY266127 | Spain | Csa | isolate |
| AY266128 | Spain | Csa | isolate |
| AY266129 | Spain | Csa | isolate |
| AY266130 | Spain | Csa | isolate |
| AY266132 | Spain | Csa | isolate |
| AY266134 | Spain | Csa | isolate |
| AY266135 | Spain | Csa | isolate |
| AY431049 | France | Cfb | cloning |
| AY431050 | France | Cfb | cloning |
| AY431051 | France | Cfb | cloning |
| AY431052 | France | Cfb | cloning |
| AY431053 | France | Cfb | cloning |
| AY431054 | France | Cfb | cloning |
| AY431055 | France | Cfb | cloning |
| AY431056 | France | Cfb | cloning |
| AY431058 | France | Cfb | cloning |
| AY431059 | France | Cfb | cloning |
| AY431060 | France | Cfb | cloning |
| AY431061 | France | Cfb | cloning |
| AY431062 | France | Cfb | cloning |
| AY431063 | France | Cfb | cloning |
| AY431064 | France | Cfb | cloning |
| AY431065 | France | Cfb | cloning |
| AY431066 | France | Cfb | cloning |
| AY431070 | France | Cfb | cloning |
| AY431071 | France | Cfb | cloning |
| AY431072 | France | Cfb | cloning |
| AY431073 | France | Cfb | cloning |
| AY431074 | France | Cfb | cloning |
| AY431075 | France | Cfb | cloning |
| AY431077 | France | Cfb | cloning |
| AY431078 | France | Cfb | cloning |
| AY431081 | France | Cfb | cloning |
| AY431086 | France | Cfb | cloning |
| AY431087 | France | Cfb | cloning |
| AY431088 | France | Cfb | cloning |
| AY431089 | France | Cfb | cloning |
| AY431090 | France | Cfb | cloning |
| AY431091 | France | Cfb | cloning |
| AY431094 | France | Cfb | cloning |
| AY431095 | France | Cfb | cloning |
| AY431097 | France | Cfb | cloning |
| AY431098 | France | Cfb | cloning |
| AY431099 | France | Cfb | cloning |
| AY672727 | Romania | Dfb | isolate |
| AY672728 | Romania | Dfb | isolate |
| AY672729 | Romania | Dfb | isolate |
| AY672732 | Romania | Dfb | isolate |
| AY672733 | Romania | Dfb | isolate |
| AY672734 | Romania | Dfb | isolate |
| AY827804 | The Netherlands | Cfb | DGGE |
| AY827827 | The Netherlands | Cfb | DGGE |
| EF116580 | China | Cfa | isolate |
| EF116581 | China | Cfa | isolate |
| EF150948 | Greece | Csa | DGGE |
| EF150951 | Greece | Csa | DGGE |
| EF150952 | Greece | Csa | DGGE |
| EF150953 | Greece | Csa | DGGE |
| EF150955 | Greece | Csa | DGGE |
| EF150958 | Greece | Csa | DGGE |
| EF150961 | Greece | Csa | DGGE |
| EF150962 | Greece | Csa | DGGE |
| EF150963 | Greece | Csa | DGGE |
| EF150964 | Greece | Csa | DGGE |
| EF150965 | Greece | Csa | DGGE |
| EF150967 | Greece | Csa | DGGE |
| EF150976 | Greece | Csa | DGGE |
| EF150977 | Greece | Csa | DGGE |
| EF150981 | Greece | Csa | DGGE |
| EF150983 | Greece | Csa | DGGE |
| EF150984 | Greece | Csa | DGGE |
| EF150986 | Greece | Csa | DGGE |
| EF150987 | Greece | Csa | DGGE |
| EF150991 | Greece | Csa | DGGE |
| EF150998 | Greece | Csa | DGGE |
| EF150999 | Greece | Csa | DGGE |
| EF151000 | Greece | Csa | DGGE |
| EF151001 | Greece | Csa | DGGE |
| EF634465 | New Zealand | Cfb | isolate |
| EF634466 | New Zealand | Cfb | isolate |
| EF634467 | New Zealand | Cfb | cloning |
| EF634468 | New Zealand | Cfb | cloning |
| EF634469 | New Zealand | Cfb | cloning |
| EF634470 | New Zealand | Cfb | cloning |
| EU233400 | Greece | Csa | DGGE |
| EU233402 | Greece | Csa | DGGE |
| AM773518 | Australia | Cfb | isolate |
| AM773521 | Australia | Cfb | isolate |
| AM773528 | USA | Dfb | isolate |
| AM773529 | USA | Dfb | isolate |
| AM773531 | Canada | Dfc | isolate |
| AM773536 | USA | Dfb | isolate |
| AM773537 | USA | Dfb | isolate |
| AM773541 | South Africa | BSh | isolate |
| AM773542 | USA | Dfb | isolate |
| HQ415607 (BG01) | Belgium | Cfb | DGGE |
| HQ415608 (BG02) | Belgium | Cfb | DGGE |
| HQ415609 (BG03) | Belgium | Cfb | cloning |
| HQ415610 (BG05) | Belgium | Cfb | cloning |
| HQ415611 (BG06) | Belgium | Cfb | cloning |
| HQ415612 (BG07) | Belgium | Cfb | cloning |
| HQ415613 (BG08) | Belgium | Cfb | cloning |
| HQ415614 (BG10) | Belgium | Cfb | cloning |
| HQ415615 (BG11) | Belgium | Cfb | cloning |
| HQ415616 (BG17) | Belgium | Cfb | cloning |
| HQ415617 (BG18) | Belgium | Cfb | cloning |
| HQ415618 (BG21) | Belgium | Cfb | cloning |
| HQ415619 (BG22) | Belgium | Cfb | cloning |
| HQ415620 (BG23) | Belgium | Cfb | cloning |
| HQ415621 (BG25) | Belgium | Cfb | cloning |
| HQ415622 (BG27) | Belgium | Cfb | cloning |
| HQ415623 (BG28) | Belgium | Cfb | cloning |
| HQ415624 (BG29) | Belgium | Cfb | cloning |
| HQ415625 (BG30) | Belgium | Cfb | cloning |
| HQ415626 (BG33) | Belgium | Cfb | cloning |
| HQ415627 (BG34) | Belgium | Cfb | cloning |
| HQ415628 (BG35) | Belgium | Cfb | cloning |
| HQ415629 (BG37) | Belgium | Cfb | cloning |
| HQ415630 (BG38) | Belgium | Cfb | cloning |
| HQ415631 (BG39) | Belgium | Cfb | cloning |
| HQ415632 (BG42) | Belgium | Cfb | cloning |
| HQ415633 (BG43) | Belgium | Cfb | cloning |
| HQ415634 (BG44) | Belgium | Cfb | cloning |
| HQ415635 (BG45) | Belgium | Cfb | cloning |
| HQ415636 (BG46) | Belgium | Cfb | cloning |
| HQ415637 (BG47) | Belgium | Cfb | cloning |
| HQ415638 (BG48) | Belgium | Cfb | cloning |
| HQ415639 (BG50) | Belgium | Cfb | cloning |
| HQ415640 (BG51) | Belgium | Cfb | cloning |
| HQ415641 (BG53) | Belgium | Cfb | isolate |
| HQ415642 (BG54) | Belgium | Cfb | isolate |
| HQ415643 (BG55) | Belgium | Cfb | isolate |
| HQ415644 (BG56) | Belgium | Cfb | isolate |
| HQ415645 (BG57) | Belgium | Cfb | isolate |
| HQ415646 (BG58) | Belgium | Cfb | isolate |
| HQ415647 (BG59) | Belgium | Cfb | isolate |
| HQ415648 (BG60) | Belgium | Cfb | isolate |
| HQ415649 (BG61) | Belgium | Cfb | isolate |
| HQ415650 (BG63) | Belgium | Cfb | isolate |
| HQ415651 (BG65) | Belgium | Cfb | DGGE |
| HQ415652 (BG66) | Belgium | Cfb | isolate |
| HQ415653 (BG68) | Belgium | Cfb | isolate |
| HQ415654 (BG70) | Belgium | Cfb | DGGE |
| HQ415655 (BG71) | Belgium | Cfb | DGGE |
| HQ415656 (BG72) | Belgium | Cfb | DGGE |
| HQ415657 (BG73) | Belgium | Cfb | DGGE |
| HQ415658 (BG74) | Belgium | Cfb | DGGE |
| HQ415659 (DG01) | Denmark | Cfb | DGGE |
| HQ415660 (DG02) | Denmark | Cfb | DGGE |
| HQ415661 (DG04) | Denmark | Cfb | DGGE |
| HQ415662 (DG05) | Denmark | Cfb | DGGE |
| HQ415663 (DG06) | Denmark | Cfb | DGGE |
| HQ415664 (NG02) | The Netherlands | Cfb | DGGE |
| HQ415665 (NG03) | The Netherlands | Cfb | DGGE |
| HQ415666 (NG04) | The Netherlands | Cfb | DGGE |
| HQ415667 (NG06) | The Netherlands | Cfb | DGGE |
| HQ415668 (NG07) | The Netherlands | Cfb | DGGE |
| HQ415669 (NG09) | The Netherlands | Cfb | DGGE |
| HQ415670 (NG10) | The Netherlands | Cfb | DGGE |
| HQ415671 (SG01) | Spain | Csa | DGGE |
| HQ415672 (SG02) | Spain | Csa | DGGE |
| HQ415673 (EG02) | Ethiopia | BSh | DGGE |
| HQ415674 (EG05) | Ethiopia | BSh | DGGE |
| HQ415675 (EG06) | Ethiopia | BSh | DGGE |
| HQ415676 (EG07) | Ethiopia | BSh | DGGE |
| HQ415677 (EG09) | Ethiopia | BSh | DGGE |
| HQ415678 (EG11) | Ethiopia | BSh | DGGE |
| HQ415679 (EG12) | Ethiopia | BSh | DGGE |
| HQ415680 (EG13) | Ethiopia | BSh | DGGE |
| HQ415681 (EG15) | Ethiopia | BSh | DGGE |
| HQ415682 (EG16) | Ethiopia | BSh | DGGE |
| HQ415683 (EG17) | Ethiopia | BSh | DGGE |
| HQ415684 (EG19) | Ethiopia | BSh | DGGE |
| HQ415685 (EG21) | Ethiopia | BSh | DGGE |
| HQ415686 (EG22) | Ethiopia | BSh | DGGE |
| HQ415687 (EG24) | Ethiopia | BSh | DGGE |
| HQ415688 (EG25) | Ethiopia | BSh | DGGE |
| HQ415689 (EG26) | Ethiopia | BSh | DGGE |
| HQ415690 (EG27) | Ethiopia | BSh | DGGE |
| HQ415691 (EG31) | Ethiopia | BSh | DGGE |
| HQ415692 (EG34) | Ethiopia | BSh | DGGE |
| HQ415693 (EG36) | Ethiopia | BSh | DGGE |
| HQ415694 (EG38) | Ethiopia | BSh | cloning |
| HQ415695 (EG39) | Ethiopia | BSh | cloning |
| HQ415696 (EG40) | Ethiopia | BSh | cloning |
| HQ415697 (EG41) | Ethiopia | BSh | cloning |
| HQ415698 (EG42) | Ethiopia | BSh | isolate |
| HQ415699 (EG43) | Ethiopia | BSh | isolate |
| HQ415700 (EG44) | Ethiopia | BSh | DGGE |
| HQ415701 (EG45) | Ethiopia | BSh | DGGE |
| HQ415702 (ArG01) | Argentina | ET | DGGE |
| HQ415703 (ArG02) | Argentina | ET | DGGE |
| HQ415704 (ArG03) | Argentina | ET | DGGE |
| HQ415705 (ArG04) | Argentina | Cfc | DGGE |
| HQ415706 (BrG01) | Brazil | Aw | DGGE |
| HQ415707 (BrG02) | Brazil | Aw | DGGE |
| HQ415708 (BrG03) | Brazil | Aw | DGGE |
| HQ415709 (BrG04) | Brazil | Aw | DGGE |
| HQ415710 (BrG05) | Brazil | Aw | DGGE |
| HQ415711 (BrG06) | Brazil | As | DGGE |
| HQ415712 (BrG07) | Brazil | As | DGGE |
| HQ415713 (BrG10) | Brazil | As | DGGE |

*For sequences obtained in this study the accession number and code is shown.
